# Supplementary figures and images for: Epigenetic dynamics of centromeres and neocentromeres in Cryptococcus deuterogattii
Source: PLoS Genet. 2021 Aug 31;17(8):e1009743. doi: 10.1371/journal.pgen.1009743 (PMC8407549; doi:10.1371/journal.pgen.1009743)

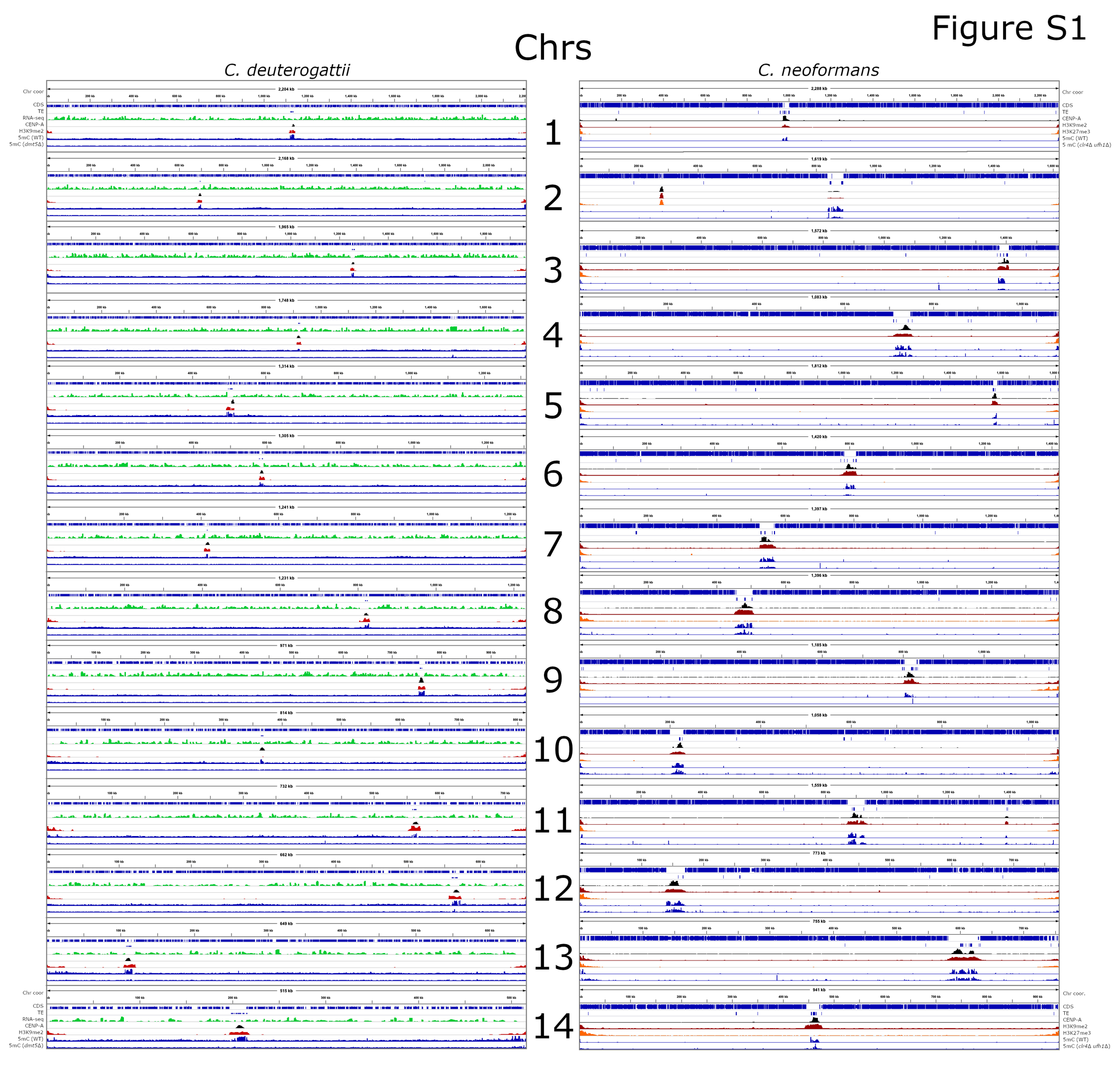

Supplement: S1 Fig — All 14 chromosomes are depicted for both C. neoformans and C. deuterogattii. For each chromosome, plots presented show the chromosome coordinates, gene content/CDS (blue), TE content (blue), RNA-seq (for C. deuterogattii only) (green), CENP-A enrichment (black), H3K9me2 enrichment (red), H3K27me3 enrichment (for C. neoformans only) (orange), and 5mC data (blue) [25]. (TIF) [file pgen.1009743.s001.tif]

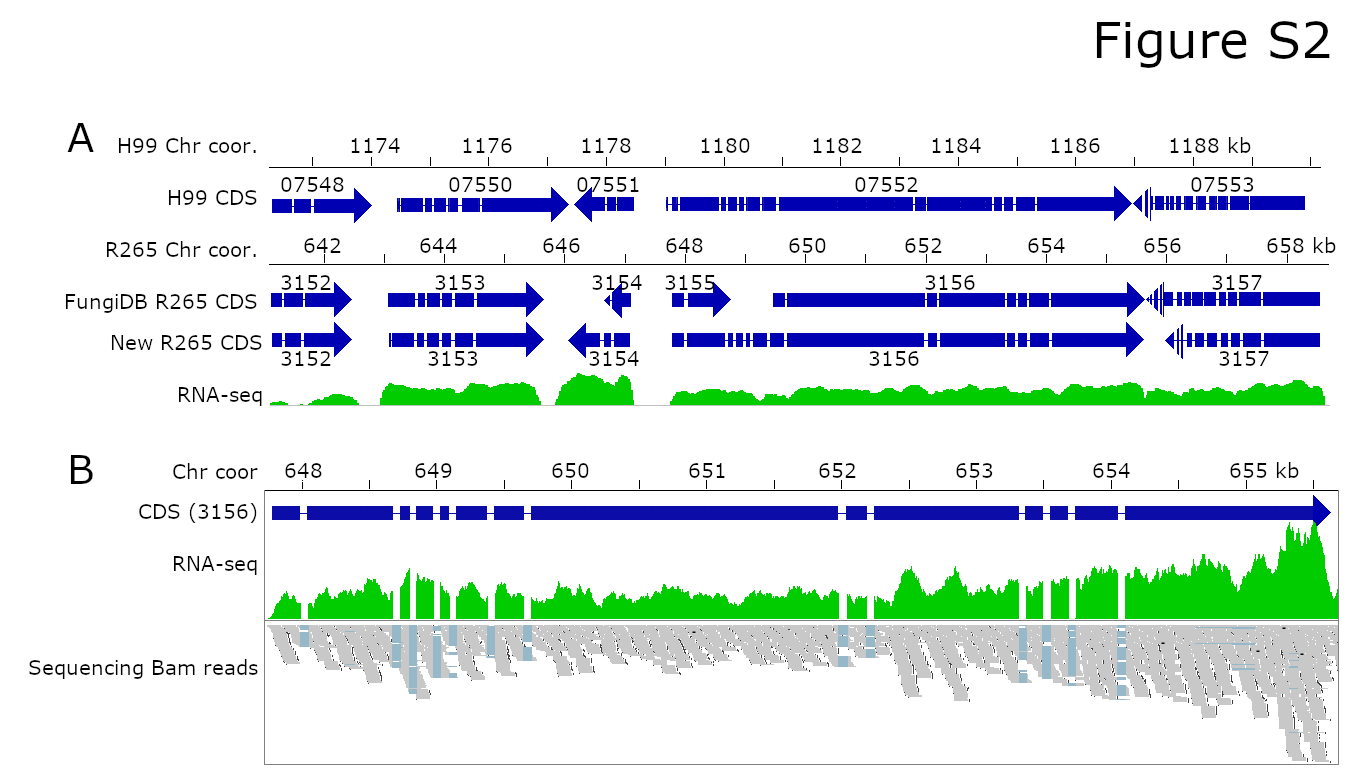

Supplement: S2 Fig — (A) Alignment view with genomic regions surrounding the DMT5 gene (CNAG_07752 and CNBG_3156) of C. neoformans and C. deuterogattii. Shown at the top of the panel are the chromosomal coordinates of C. neoformans. The genes are indicated with blue arrows with exons and introns marked. For C. deuterogattii, two gene annotations are shown. The old (FungiDB) annotation predicted that DMT5 was truncated. The new genome annotation shows that the DMT5 gene is full-length and has a similar length to the C. neoformans ortholog [41]. (B) Detailed view of C. deuterogattii introns and exons of the DMT5 gene is supported by RNA-seq reads, which are shown to support the gene structure. (TIF) [file pgen.1009743.s002.tif]

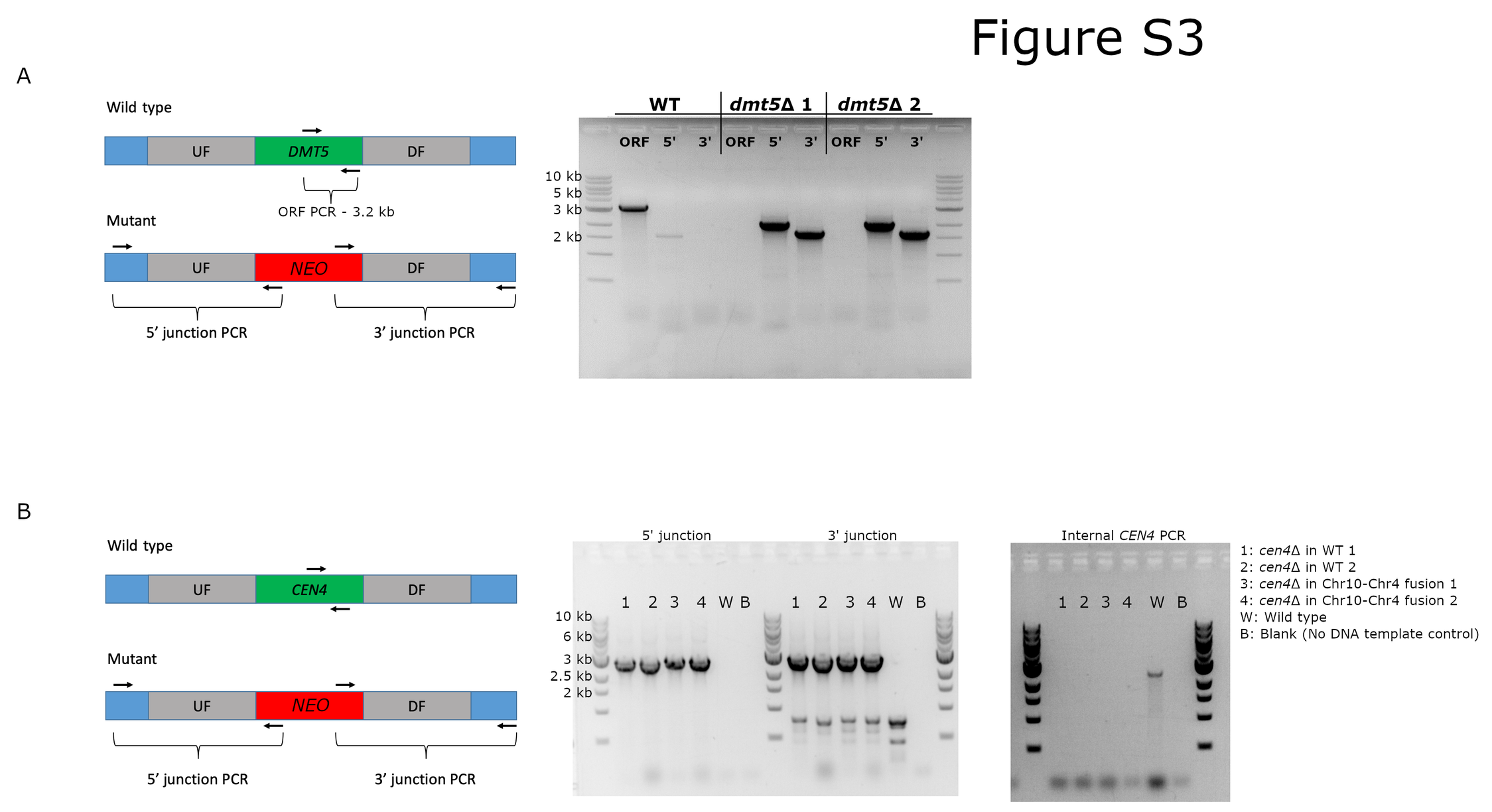

Supplement: S3 Fig — PCR confirmations for dmt5Δ and cen4Δ mutants are shown. Panel (A) shows PCR confirmations for dmt5Δ; panel (B) shows PCR confirmations for cen4Δ. For both mutants, the 5’ and 3’ junction, as well as internal/ORF PCRs, are shown. (TIF) [file pgen.1009743.s003.tif]

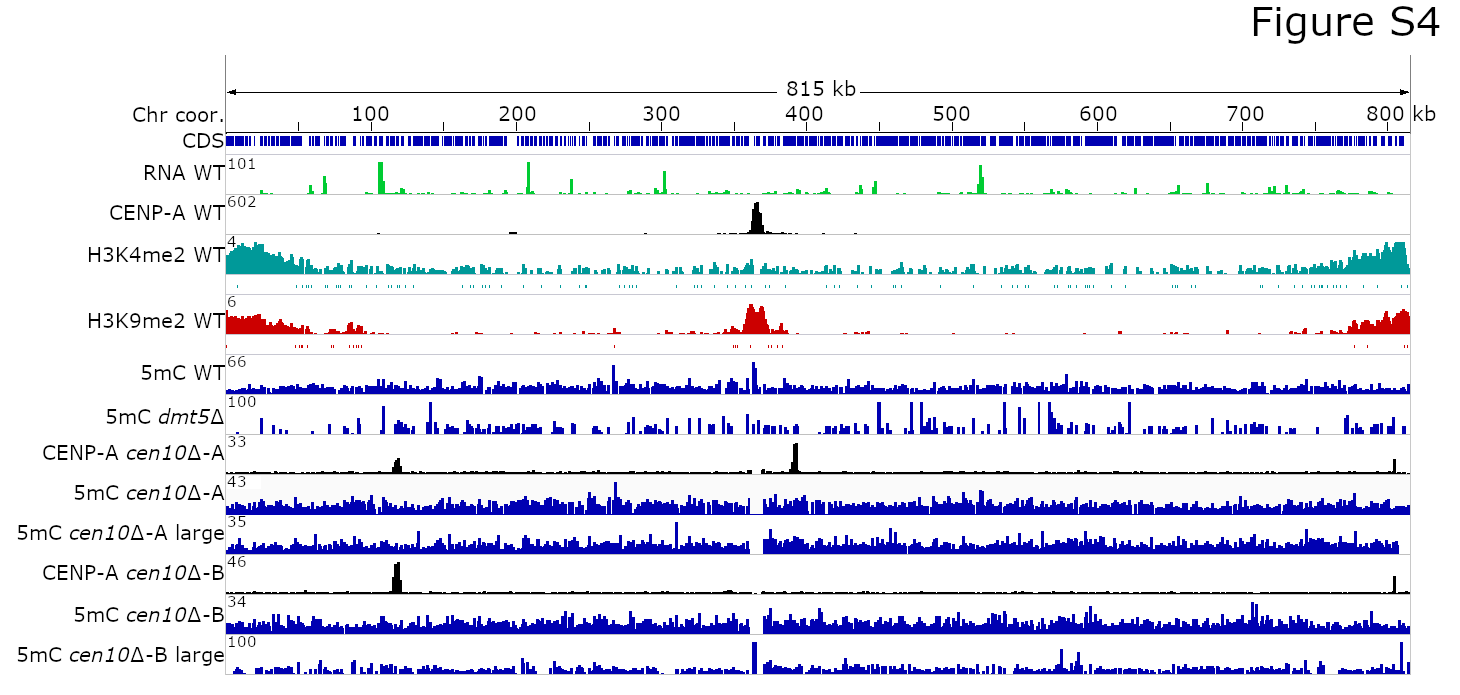

Supplement: S4 Fig — ONT sequencing was performed for two previously obtained cen10Δ and the derived chromosome fusions (large) that have silenced neocentromeres. The complete chromosome 10 is shown and chromosomal coordinates are indicated. The wild-type genome annotation (CDS), RNA-seq (green), CENP-A ChIP-seq (black), H3K4me2 ChIP-seq (turquoise) and ChIP-seq for H3K9me2 (red) is shown. DNA methylation analyses based on ONT sequencing are shown in blue for WT, dmt5Δ, and cen10Δ mutants -A and -B and their derived chromosome fusion products. For each neocentromere mutant, the CENP-A ChIP-seq track shows the neocentromere location. (TIF) [file pgen.1009743.s004.tif]

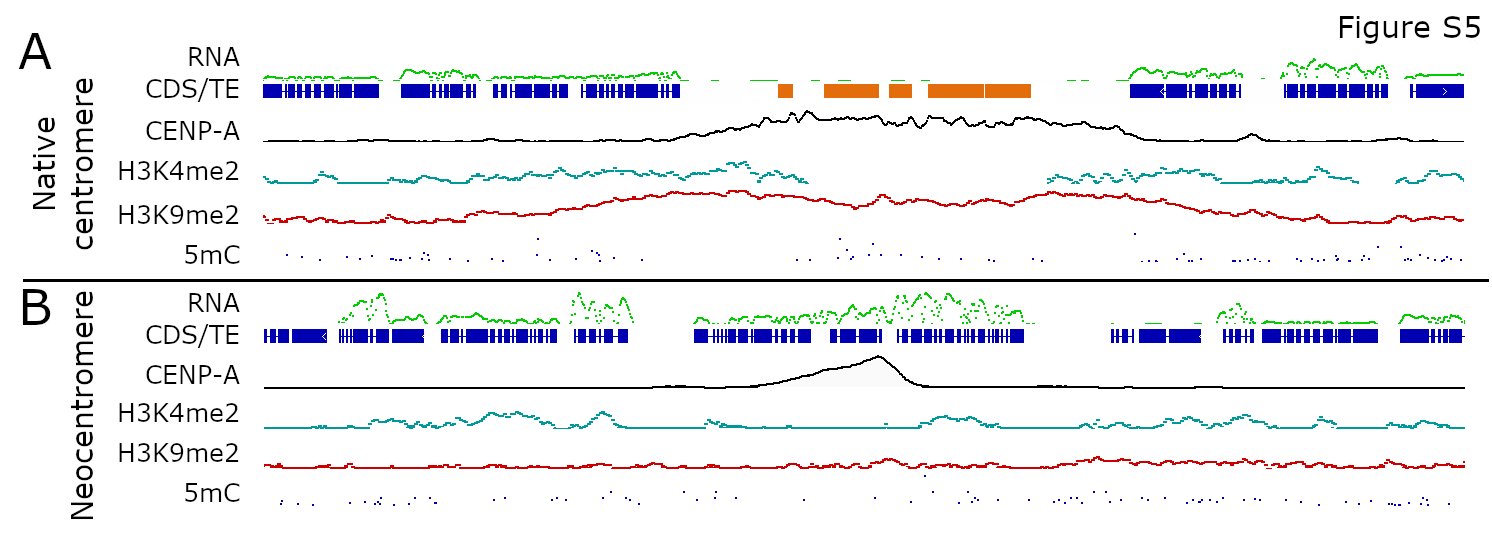

Supplement: S5 Fig — Panel (A) shows the organization of a native centromere. Native centromeres are located in ~14.5 kb ORF-free chromosomal regions and these regions are enriched for truncated transposable elements (TE, orange). The whole centromeric region is modestly enriched for 5mC DNA methylation (blue), enriched for H3K9me2 (red) and within this heterochromatic region, the CENP-A peak (Black) is located. The centromeric regions are flanked by genes (dark blue) that are actively expressed (green) and enriched for the euchromatic histone mark H3K4me2 (green). Panel (B) shows an example of a neocentromere. Neocentromeres span actively expressed (green) genes (blue), are determined by the presence of CENP-A (black) and lacked any enrichment for the epigenetic marks that were analyzed, while the genes are still enriched for H3K4me2 (green). (TIF) [file pgen.1009743.s005.tif]

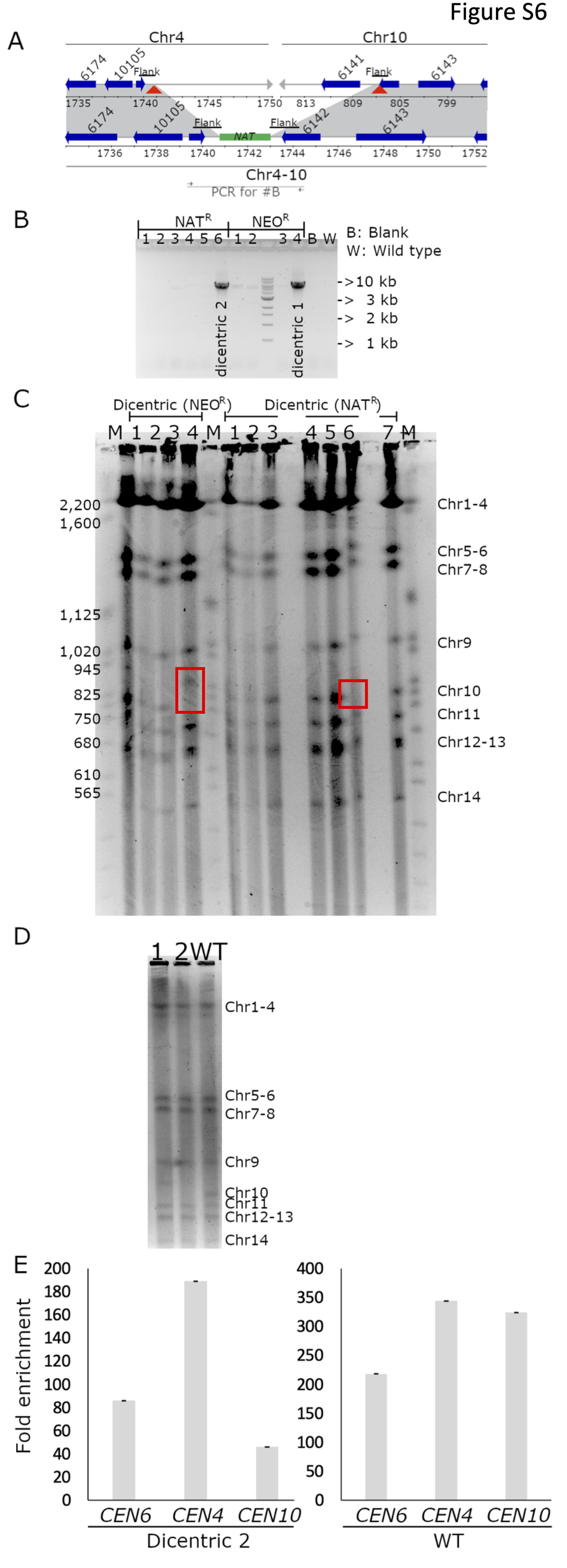

Supplement: S6 Fig — (A) Schematic view of the formation of a dicentric chromosome. At the top, a sub-telomeric region of wild-type Chr4 and Chr10 is shown. The chromosomal targets of the guide RNAs are depicted by red triangles. The double-stranded DNA breaks were repaired by homologous recombination that was mediated by an overlap PCR product containing regions homologous to both chromosomes flanking a selectable marker. The homologous regions are depicted by black lines and labeled “Flank”. (B) Spanning PCR analysis confirmed chromosome fusion in mutant 1 (G418R 4) and mutant 2 (NatR 6) are shown. The region amplified for the spanning PCR is indicated in panel A with a bar labeled with “PCR for #B”. (C) PFGE analysis with all mutants obtained after recovering mutants on selective media. Dicentric 1 (G418R 4) and 2 (NatR 6) lack a wild-type size band for Chr10 confirming the spanning PCR products in panel A and these are indicated with a red rectangle. (D) PFGE analysis shows that wild-type Chr 10 is absent in dicentric strains 1 and 2, which confirms that Chr4 and Chr10 are fused in these two strains. Instead of the wild-type Chr10 band, mutant dicentric 1 has an additional band (~60 kb higher) than the wild-type Chr10 band. Based on this PFGE, dicentric 1 has no additional resolvable bands. (E) ChIP-qPCR analyses were performed for dicentric mutant 2 and wild type. CENP-A fold enrichment is shown for CEN4 and CEN10 and a positive control (CEN6). The fold-enrichment was compared to actin as the negative control. (TIF) [file pgen.1009743.s006.tif]

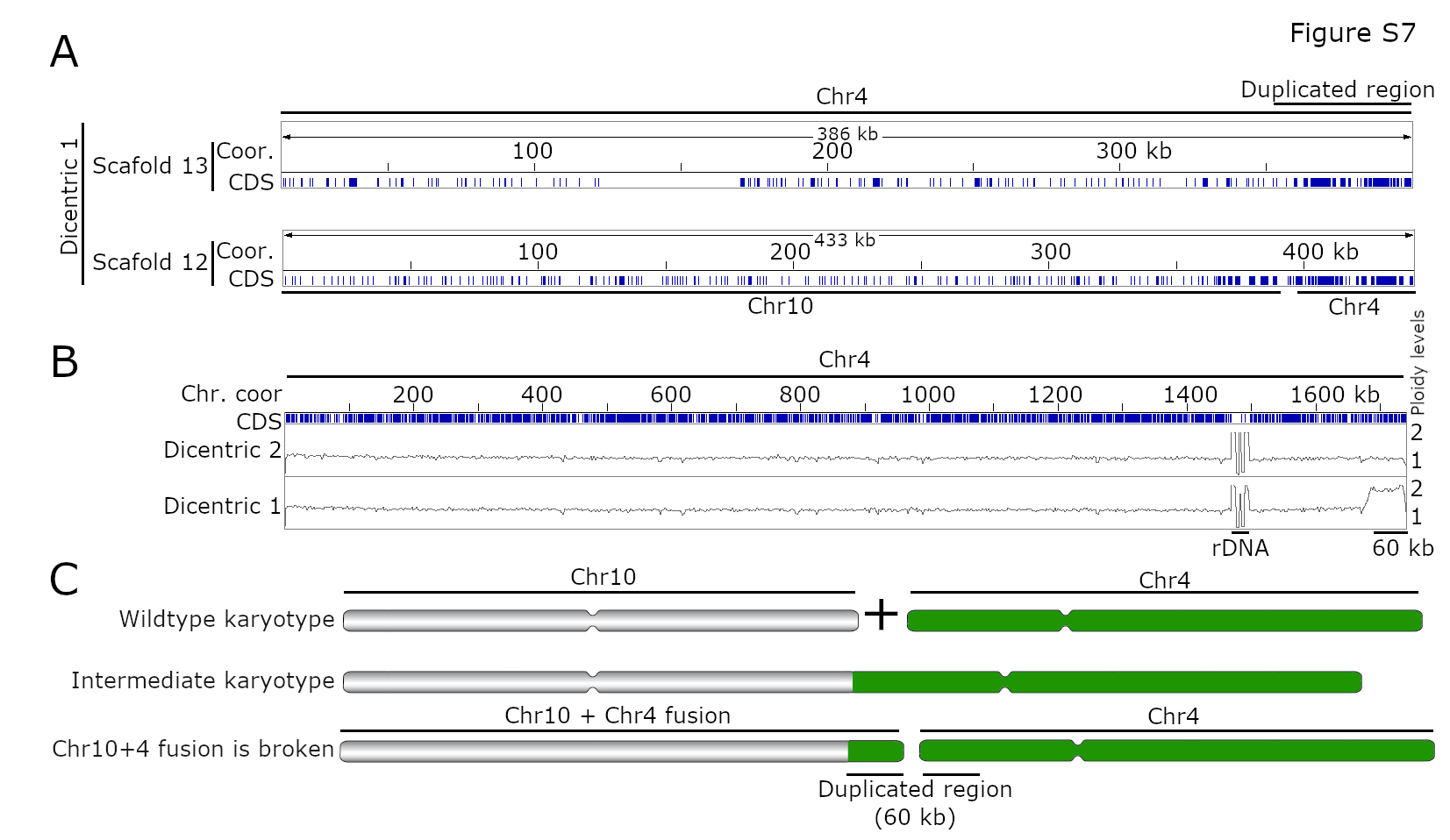

Supplement: S7 Fig — Panel (A) shows the two scaffolds that harbor the ~60 kb duplicated region. Scaffold 13 corresponds to a part of chromosome 4, and scaffold 12 is the broken chromosomal fusion product of chromosome 10 and 4. (B) The panel shows Illumina sequencing of the dicentric mutants mapped to the wild-type genome. Short read sequencing indicated that a ~60 kb region of chromosome 4 is duplicated in dicentric 1 and has a ploidy level of two as compared to the rest of the chromosome. As a control, dicentric isolate 2 was sequenced and this strain has a ploidy level of one for the entire chromosome 4. (C) Putative model explaining the chromosome fusion between chromosome 4 and 10, the intermediate state in which the fused chromosome is intact, and the final karyotype after the chromosomal breakage for dicentric 1. (TIF) [file pgen.1009743.s007.tif]

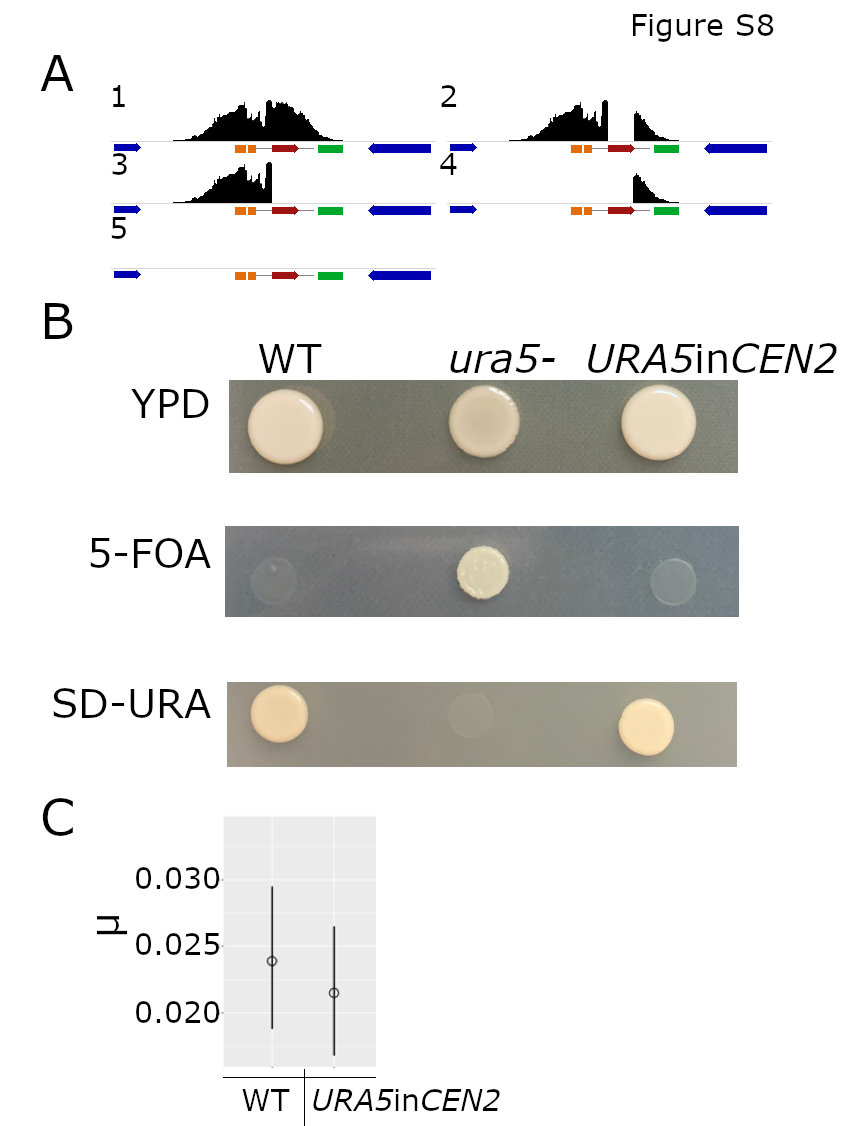

Supplement: S8 Fig — (A) A schematic model of CEN2 is shown. The CENP-A peak is shown in black, genes flanking the pericentric region are shown in blue. The URA5 gene is shown in red and the truncated transposable elements are shown in orange and green (Tcn4 and Tcn6). Hypothetical outcomes for URA5 integration into CEN2 include: (1) CENP-A would cover the URA5 gene making the CENP-A-bound region larger than the native centromere; (2) URA5 gene might divide the CENP-A-enriched region into two independent regions; (3 & 4) either one of the regions flanking URA5 would be enriched for CENP-A, generating a smaller centromere; (5) URA5 integration abolishes CEN2 function leading to neocentromere formation. (B) Prior to the ChIP-seq experiment for URA5inCEN2, the strain was tested for URA5 expression. For all three plates, the wild type was included as a control, and ura5- is the parental strain in which the URA5 gene was integrated into CEN2. As expected all three strains grew on the control medium (YPD). Only ura5- strain was able to grow on a medium containing 5-FOA. On the SD-uracil (SD-URA) medium, the wild-type and URA5inCEN2 strains were able to grow due to the presence of an active URA5 gene. (C) Assay shows that the URA5 gene in wild-type and URA5inCEN2 mutant has a similar mutation rate when growing on YPD or 5-FOA medium. The mutation rate (μ) is shown on the y-axis. (TIF) [file pgen.1009743.s008.tif]

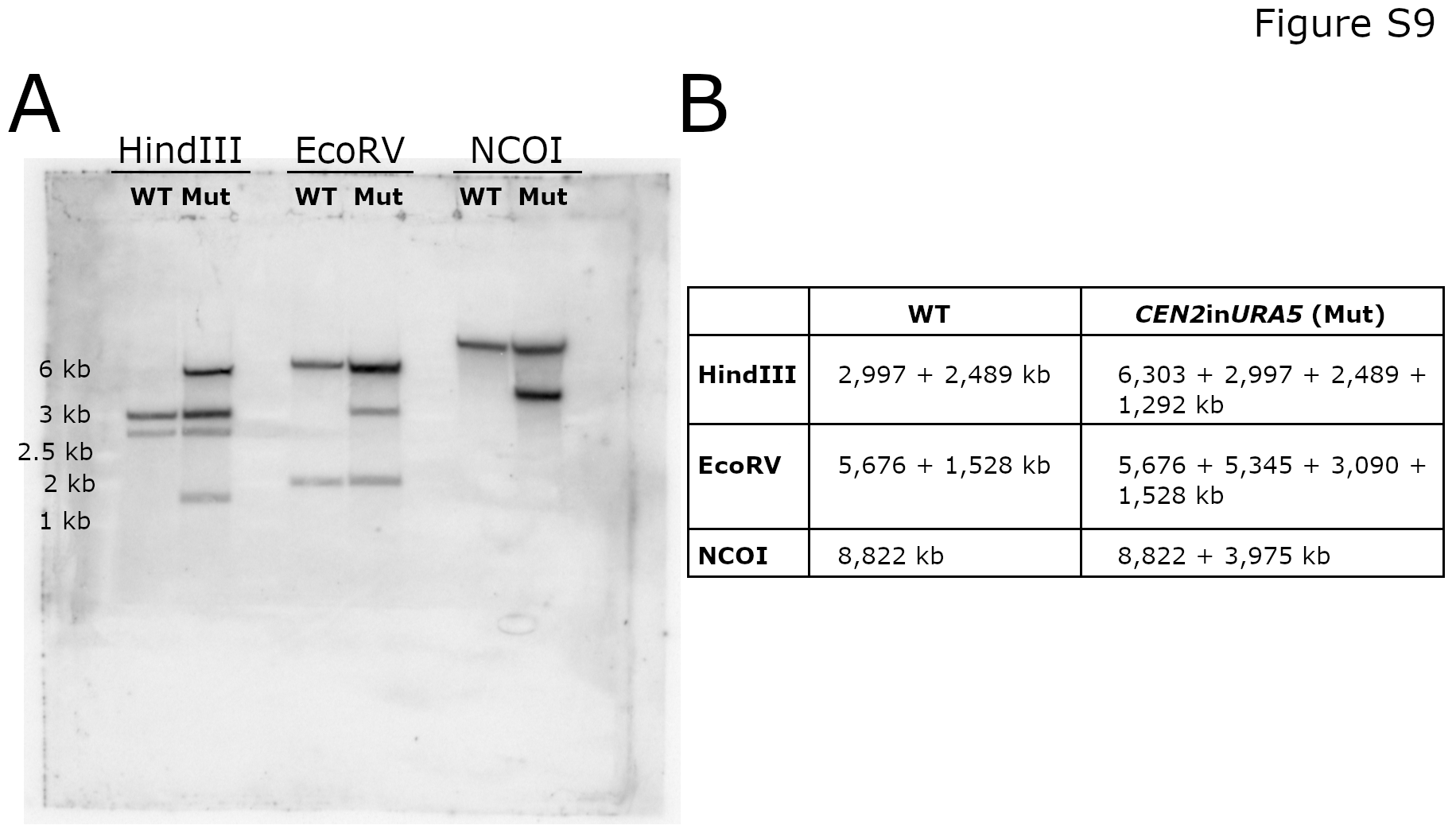

Supplement: S9 Fig — (A) Southern blot analysis with three independent restriction digest analyses of the CEN2inURA5 strain. The URA5 gene sequence was used as a probe. The Southern blot hybridization pattern confirms that a single copy of the URA5 gene has been inserted at the desired targeted site in CEN2. (B) Table indicating the expected restriction digest product sizes. Because the CEN2inURA5 strain still has the ura5- gene present at its native locus, the pattern shows products from both the ura5 native gene and the CEN2inURA5 transgene. (TIF) [file pgen.1009743.s009.tif]
