# Supplementary material for: Epigenetic dynamics of centromeres and neocentromeres in Cryptococcus deuterogattii
Source: PLoS Genet. 2021 Aug 31;17(8):e1009743. doi: 10.1371/journal.pgen.1009743 (PMC8407549; doi:10.1371/journal.pgen.1009743)
Supplement: S1 Text — (DOCX) [file pgen.1009743.s010.docx]

**ChIP-seq**

**Quality control**

fastx_clipper -C -M 40 -a *adapter_sequence*-v -i *sample*.fastq -o *sample*_adapter.fastq

fastx_trimmer -Q33 -f5 -v -i *sample*_adapter.fastq -o *sample*_adapter_trim.fastq

fastq_quality_filter -Q33 -v -q 20 -p 80 -i *sample*_adapter_trim.fastq -o *sample*_adapter_trim_quality.fastq

**Bowtie2**

bowtie2-build -f *genome*.fa *genome*_reference

bowtie2 -p 2 -x *genome*_reference -1 *sample1*.fastq -2 *sample2*.fastq -S *sample*_bowtie.sam

**Samtools**

samtools faidx *genome*.fa

for f in *.sam ; do samtools view -S -b -@ 10 $f | samtools sort -o `basename $f .sam`.sorted.bam ; done && for f in *.sorted.bam ; do samtools index $f ; done && for f in *.sorted.bam ; do samtools flagstat $f > `basename $f .sorted.bam`.sorted.bam.flagstat ; done

**Subtract sample - input**

bamCompare -b1 *sample*.sorted.bam -b2 *input*.sorted.bam -o *sample-input*.bw

**IGV tools**

for f in *.sorted.bam ; do igvtools count -z 7 -w 25 --minMapQuality 0 --windowFunctions mean --pairs $f $f.z7.w0.mQ60.wFmean.Pairs.tdf *.fa ; done

**Peak calling with MACS2**

macs2 callpeak -t *sample*.sorted.bam -c *input*.sorted.bam -f BAM -g mm -n *sample* -B -q 0.01 --outdir MACS

**RNA-seq**

**Hisat2**

Hisat2 was followed according standard settings as was descript in the Hisat2 publication.

﻿

Extract splice-site and exon information from the gff file:

extract_splice_sites.py *annotation*.gff > splice.ss

extract_exons.py *annotation.*gff > exon.exon

Build a HISAT2 index:

hisat2-build --ss splice.ss --exon exon.exon *genome*.fa *genome*_tran

﻿

Align the rna-seq reads to the genome:

hisat2 -p 8 --dta -x *genome_tran* -1 *sample*.fastq.gz -2 *sample*.fastq.gz -S *sample_genome*.sam

﻿

Sort and convert the SAM files to BAM:

samtools sort -@ 8 -o *sample_genome*.bam *sample_genome*.sam

﻿Assemble transcripts for each sample:

stringtie -p 8 -G *annotation.*gff -o *RNA-seq_annotation.*gtf –l *sample_genome*.bam

﻿Examine how the transcripts compare with the reference annotation (optional):

gffcompare –r *annotation*.gtf –G –o *RNA-seq_annotation.*gtf
